# Emotional responses to climate change in Norway and Ireland: a validation of the Inventory of Climate Emotions (ICE) in two European countries and an inspection of its nomological span

**DOI:** 10.3389/fpsyg.2024.1211272

**Published:** 2024-02-08

**Authors:** Michalina Marczak, Małgorzata Wierzba, Bartosz Kossowski, Artur Marchewka, Roxanna Morote, Christian A. Klöckner

**Affiliations:** ^1^Department of Psychology, Norwegian University of Science and Technology, Trondheim, Norway; ^2^Laboratory of Brain Imaging, Nencki Institute of Experimental Biology, Polish Academy of Sciences, Warsaw, Poland

**Keywords:** emotional responses, climate change, inventory of climate emotions (ICE), cross-cultural validity, climate anger, climate anxiety, climate guilt

## Abstract

There is an increasing research interest in emotional responses to climate change and their role in climate action and psycho-social impacts of climate change. At the same time, emotional experience of climate change is multidimensional and influenced by a variety of factors, including the local cultural context. Here, we contribute to the scientific debate about this topic with original quality-controlled data from the general populations in Norway (*N* = 491) and Ireland (*N* = 485). We investigate the cross-cultural validity and the nomological span of eight distinct emotional responses to climate change - climate anger, climate contempt, climate enthusiasm, climate powerlessness, climate guilt, climate isolation, climate anxiety, and climate sorrow - measured using the recently introduced Inventory of Climate Emotions. We first validate the 8-factor structure of the Norwegian and English language versions of the ICE. Subsequently, we demonstrate a high degree of cross-cultural measurement invariance for these eight climate emotions. Finally, we explore the relationships between these emotional responses and a range of theoretically relevant variables. In this final step, we show that climate emotions are differentially linked to climate change perceptions, support for mitigation policies, socio-demographic factors, feelings of loneliness and alienation, environmental activism, and the willingness to prioritize the natural environment over one’s immediate self-interests. Some of these links are also differentiated by the cultural context. This research presents further evidence for the structural, cross-cultural, and concurrent validity of climate emotions as postulated in the ICE framework. Moreover, it provides tools in the form of validated Norwegian and English language versions of the ICE, the complete R code for the validation analysis, as well as an informed basis for cross-cultural research on emotional responses to climate change.

## Introduction

Given the urgency of the climate crisis (IPCC, 2022) and the affective power of emotions to shape the way people operate in the world (Riedner, 2006; Berlant, 2011; Massumi, 2015; Dukes et al., 2021), there is an increasing research focus on the role of emotional responses to climate change in pro-climate action and psycho-social impacts of climate change (e.g., Clayton, 2020; Brosch and Steg, 2021; Wullenkord et al., 2021; Sangervo et al., 2022). Deliberately and actively incorporating people’s emotions in efforts for sustainability transition has been demonstrated to be effective in addressing psychological hurdles that often lead to apathy and disengagement in the face of the environmental crisis (Morris et al., 2019; Van Valkengoed and Steg, 2019; Xie et al., 2019; Schneider et al., 2021). Thus, understanding climate emotions may hold a key to enhancing individuals’ receptiveness of this issue and inspiring pro-environmental efforts (Brosch, 2021).

Much of the existing research in this regard has focused on the issue of anxiety in the context of global environmental change. This topic has been approached from various perspectives, including through the socio-political, existential, psychodynamic, and psychopathological lens (Pihkala, 2020). A significant body of psychological research has primarily concentrated on the latter, discussing the links between adverse emotions related to climate change and decreased mental well-being (e.g., Searle and Gow, 2010; Helm et al., 2018; Clayton and Karazsia, 2020; Hogg et al., 2021; Innocenti et al., 2021; Ogunbode et al., 2021, 2022; Reyes et al., 2021). In this vein, a series of investigations to explore the nature of eco-anxiety showed that while habitual worry about climate change may be unconstructive and indicative of psychological suffering for some individuals, it is, for many others, a constructive, adaptive pro-environmental response (Verplanken et al., 2020). These findings suggest that strong emotional responses to climate change are intertwined with the motivation to engage in pro-environmental actions. In addition, it is worth highlighting that, although certain individuals do experience psychological distress as a reaction to the environmental crisis (Clayton and Karazsia, 2020; Innocenti et al., 2021; Ogunbode et al., 2021; Reyes et al., 2021), the proportion of those grappling with such distressing anxiety appears relatively limited when compared to the broader population expressing concern about the state of the environment (Hajek and König, 2022; Whitmarsh et al., 2022). Bearing these observations in mind, there is a need to consider a wider range of emotions in response to the environmental crisis.

In this context, nationally representative surveys have uncovered a wide array of emotional responses related to climate change. To illustrate, in a survey conducted in the United States using closed-ended questions, it was found that 62% of Americans associated global warming with a combination of worry and interest. Furthermore, approximately 40% of respondents linked it to a range of emotions, including disgust, helplessness, hopefulness, anger, and fear (Leiserowitz et al., 2018). In a similar study conducted in Greenland, approximately 40% of participants reported experiencing moderate to intense feelings of hopefulness and fear concerning climate change, while 30% expressed happiness. Around 20% of respondents reported emotions like sadness, disgust, hopelessness, guilt, or anger (Minor et al., 2019).

Likewise, Glenn Albrecht, a pioneering figure in the field of emotional responses to environmental harm, developed a framework encompassing a wide spectrum of mental and emotional states linked to the specific circumstances of one’s natural surroundings (Albrecht, 2019). Building on his previously developed concept of “solastalgia,” which aimed to encapsulate the emotions associated with the loss of a nurturing environment, as well as the feelings of environmental injustice and powerlessness experienced when one’s home environment undergoes imposed transformation (Connor et al., 2004; Albrecht et al., 2007), he introduced a range of other eco-emotions. These include, e.g., “ecoparalysis,” which denotes a sense of powerlessness in the face of the overwhelming environmental crisis, or “terrafurie,” which signifies intense anger directed specifically at those who hold the power to cause harm to the Earth (Albrecht, 2019).

Positive emotions in the context of impending climate change were investigated in detail by Maria Ojala, who made a notable distinction between two forms of hope: *constructive hope*, characterized by positive sentiments about the future stemming from a positive reassessment of the situation, confidence in various societal actors, and faith in the effectiveness of personal actions, as opposed to *hope rooted in denial* (Ojala, 2012). Additionally, positive emotions have been shown to be linked to environmentally-friendly behavior (Taufik et al., 2015; Venhoeven et al., 2016; Zawadzki et al., 2020).

In sum, an increasing body of research has demonstrated that emotional experience of climate change is comprised of a wide panorama of emotions that differentially affect people’s mental wellbeing and pro-climate behavior (e.g., Kleres and Wettergren, 2017; Wang et al., 2018; Caillaud et al., 2019; Stanley et al., 2021; Pihkala, 2022; Marczak et al., 2023b). In this paper, we aim to investigate multiple emotional responses to climate change in more detail, paying special attention to the issues of valid measurement of climate emotions in the cross-cultural perspective.

### Measurement of climate emotions

Climate emotions research is a dynamic field with various measurement approaches. First of all, several questionnaires were developed and psychometrically validated in research on emotional impacts of climate change in relation to mental distress [e.g., the Environmental Distress Scale (Higginbotham et al., 2007), Climate Anxiety Scale (Clayton and Karazsia, 2020), Hogg Eco-Anxiety Scale (Hogg et al., 2021)]. While these questionnaires include affective components, their primary focus lies in evaluating mental distress associated with climate change. Simultaneously, research examining the fundamental emotional aspects of climate change, beyond the scope of mental health, depended principally on compilations of emotion-related terminology, which exhibited considerable variations across studies (Marczak et al., 2023a). One challenge associated with utilizing emotion-word lists is that single items are more susceptible to random measurement errors and undisclosed biases in meaning and interpretation, as compared to multi-item scales (Bergkvist and Rossiter, 2007; Furr, 2011).

Against this background, recently, several multi-item operationalizations of climate emotions have emerged. Galway and Beery (2022) built upon the list of emotion-words proposed in the Yale Program on Climate Change Communication (Leiserowitz et al., 2018) to investigate emotional responses to climate change in Canada’s Provincial North. Their measure, referred to as the Climate Emotion Scale (CES), encompassed 11 items, each referring to a distinct emotion, including worry, frustration, sadness, helplessness, fear, anger, hopelessness, hope, anxiety, resilience, and guilt. Confirmatory factor analysis of these items indicated that they could be categorized into two factors: positive and negative emotions. Furthermore, the high internal consistency of all the items taken together implied the importance of considering the diversity of climate emotions in an integrated manner, a concept the authors labeled as the ‘constellation of climate emotions’.” As such, the CES evaluates what emotions people report to feel when they are asked to think of climate change. In this sense, it does not measure the specifics of various climate emotions.

Ágoston et al. (2022) adopted a different approach, combining a literature review and qualitative analysis of semi-structured interviews to create multi-item scales for measuring three distinct eco-emotions: eco-anxiety, eco-guilt, and ecological grief. The scales were then psychometrically validated in the Hungarian context and moderate to strong correlations were identified among them, indicating connections between the constructs, while also emphasizing the independence of each emotion. This was further supported by varying associations between different eco-emotions and pro-environmental behaviors. These findings highlight the importance of individually assessing different climate emotions, as they are likely to have diverse implications for pro-environmental engagement.

Concurrently, based on their research conducted in Poland, Marczak et al. (2023a) proposed measuring a yet wider array of emotional responses to climate change across eight dimensions systematized in the Inventory of Climate Emotions (ICE). In their framework, each factor represented emotional experience accompanying specific perceptions of climate change. The content of the ICE was informed by extensive in-depth exploratory research, literature review, and expert content validation. The first dimension, *climate anger*, captures the resentment around the perception that people in power have not been doing enough to mitigate climate change or that they have been intentionally harming the climate. *Climate contempt* refers to the feelings around having disregard for the issue of climate change. *Climate enthusiasm* captures positive feelings around addressing climate change such as hopefulness and a sense of vigor around collective climate action. *Climate powerlessness* relates to the feelings of helplessness around the perception that one has little individual agency to fight climate change. *Climate guilt* captures the remorse and culpability around the perception that one’s behavior has a negative impact on the climate. *Climate isolation* describes the feelings of loneliness around the perception that other people are not engaged enough in the topic of climate change. *Climate anxiety* captures both apprehension and a sense of hopelessness around one’s very negative view of the future under the progressing climate change. Finally, *climate sorrow* refers to sadness due to the perception that climate change is causing irretrievable losses to life on Earth^1^;

Looking at the ICE framework in detail, it becomes evident that the object of emotional responses is not climate change *per se* but rather related issues. The paper presenting the development of the ICE (Marczak et al., 2023a), provided sound justification of such an approach. It argued that the fact that both climate change and human emotions are inherently multifaceted presents challenges for creating all-encompassing definitions and measures of emotional experience of climate change. In this light, grouping emotions around their specific evocative themes, that emerged in a data-driven way, allowed to establish a framework that can be consistently applied across a wider range of participants in a valid and reliable manner, as demonstrated in the validation of the ICE in Poland (Marczak et al., 2023a). Finally, the conceptualization of climate emotions as composite constructs built upon the interaction between emotions and cognitions is in line with the psychological constructionist understanding of emotion, supported by recent developments in affective science (Feldman Barrett, 2017). In summary, the ICE offers a consistent systematization of the interconnections between a spectrum of key perceptions of climate change and characteristic bundles of emotions surrounding them. In this perspective, the relationships observed between climate emotions and other variables result from a blend of emotional responses tied to the evocative themes. In effect, the ICE offers a nuanced exploration of the selected eight climate emotions, distinguishing it as a comprehensive measure for understanding a wide array of emotional responses to climate change. However, its validity beyond the Polish context has not been investigated.

### Cross-cultural generalisability of climate emotions

It is increasingly evident that emotional responses to climate change are multifaceted and shaped by a multitude of factors. A systematic review of qualitative literature revealed various dimensions of emotional responses across different regions (Soutar and Wand, 2022). For instance, the perception of climate change as a future threat was more prominent in Western countries, while populations in developing countries or disadvantaged socioeconomic groups exhibited different patterns of emotional responses, highlighting the profound impact of local context and vulnerability on climate emotions (Soutar and Wand, 2022). Moreover, an analysis of qualitative data from four island countries, namely Fiji, Cyprus, New Zealand, and the United Kingdom demonstrated that the emotional impact of climate change is influenced not only by the specific local and regional dynamics that define the experience of climate change in different places, but also by gender (Du Bray et al., 2019). This research highlighted that living in locations that are biophysically vulnerable to the effects of climate change produces significant emotional responses for everyone, even if it is displayed more as sadness among women and anger among men. The heterogeneity in the emotional perception of climate change across the world, as highlighted by these studies, presents a challenge for research in the field of climate emotions. To understand the intersection of climate change, emotions, and cultural factors, it is imperative to conduct more extensive cross-cultural research, acknowledging the diverse emotional geographies that shape people’s responses to climate change. In addition, emotions are socially organized and situated phenomena (Ahmed, 2013; Turner and Trucano, 2014), and culture and language play a fundamental role in constituting people’s emotional experience (Lindquist and Feldman Barrett, 2008; Lindquist et al., 2016).

To gain a better understanding of emotional responses to climate change and their role in climate engagement, rigorous cross-cultural research is needed. Thus, we aimed to investigate the validity of the ICE framework in two novel cultural settings - Norway and Ireland. To this end, we inspected the psychometric properties of the carefully translated/back-translated Norwegian and English-language versions of the ICE. Subsequently, to examine the stability of the theoretical structure and psychological meaning of the ICE scales across the studied cultures, we also conducted a hierarchically ordered set of tests for measurement equivalence of the ICE across three different languages and cultural contexts - Norway, Ireland and Poland.

### Nomological span of climate emotions

Besides testing for structure stability and measurement equivalence, to prove construct validity, it is critically important to establish a sound nomological span of the measured constructs (Cronbach and Meehl, 1955). Research utilizing the ICE in Poland showed that different dimensions of climate emotions captured with the instrument are meaningfully associated with a number of theoretically relevant variables (Marczak et al., 2023a). Because of their links with pro-climate behavior, attitudes, and perceptions, climate enthusiasm, anger, anxiety, and sorrow were coined the “core pro-climate emotions,” with climate guilt, isolation, and powerlessness playing a more ambiguous role in pro-climate engagement. In addition, the triad comprising climate guilt, isolation, and anxiety was strongly positively associated with the affective aspects of climate and eco-anxiety. At the same time, expectedly, climate contempt was consistently negatively associated with pro-climate engagement.

In this paper, we investigated the robustness of the nomological span of climate emotions using a partially novel set of theoretically relevant variables (Cronbach and Meehl, 1955; Crandall and Sherman, 2016). To begin with, adopting the empirical guidelines for interpreting the magnitude of correlation coefficients in psychological studies (Hemphill, 2003), we expected to directly replicate Marczak et al.’ (2023a) findings. First in H1, we assumed that *pro-climate change-perceptions are:*

- *strongly positively correlated with climate anger, climate anxiety, and climate sorrow;*
- *moderately positively correlated with climate enthusiasm, climate guilt, and climate isolation;*
- *strongly negatively correlated with climate contempt;*
- *uncorrelated with climate powerlessness*.

Moreover, in H2, we hypothesized that *support for pro-climate policies is:*

- *strongly positively correlated with climate anger, climate sorrow, climate anxiety, and climate enthusiasm;*
- *moderately positively correlated with climate guilt;*
- *strongly negatively correlated with climate contempt;*
- *uncorrelated with climate powerlessness.*

**Figure 1:**
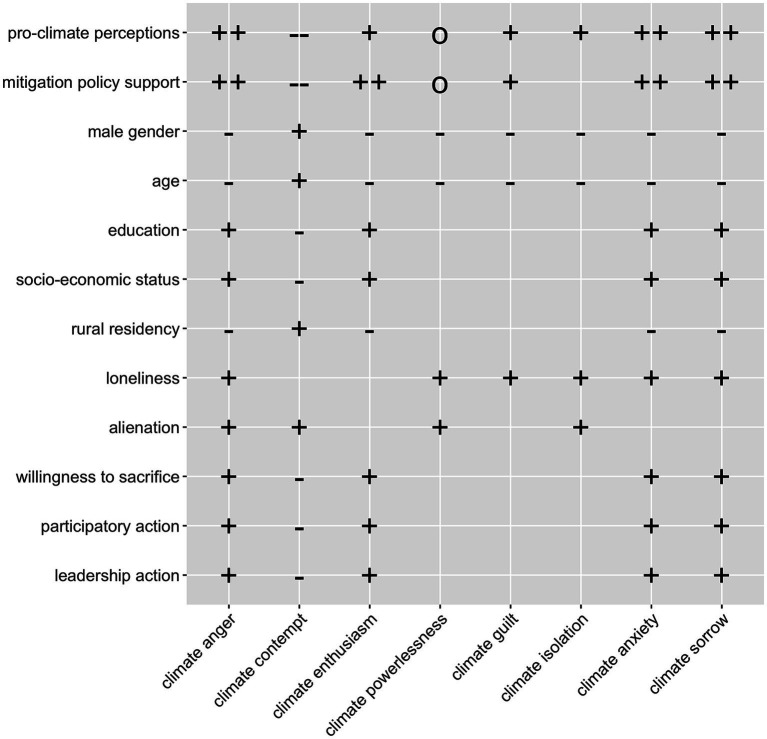
Graphical summary of the hypotheses. + positive correlation (for H1 and H2: moderate positive correlation); ++ strong positive correlation; − negative correlation;—-strong negative correlation; 0 no correlation; empty space means that there was no specific hypothesis.

In addition, we proposed that the eight climate emotions are linked to several theoretically relevant variables that have not yet been systematically studied as correlates of emotional responses to climate change. Three goals guided the selection of the variables included in this study. First, we intended to move beyond the purely psychological factors and explore the broader sociodemographic context of climate emotions. Therefore, we took a closer look at the links between variables such as gender, age, area of residence, education, and perceived socio-economic status, and the emotional experience of climate change. Second, we assessed participants’ levels of loneliness and alienation in order to quantitatively inspect the links between these two variables and climate emotions put forward in qualitative research (Marczak et al., 2023b). Finally, to extend the functional validity of the ICE in the context of pro-environmental behavior, we assessed its links with willingness to make sacrifices for the environment and with pro-environmental activism. Below, we introduce the investigated correlates in more detail and present the hypotheses. Since these analyses were partly exploratory, we did not assume the size of the effects but merely the direction of the correlations. All the hypothesized links are summarized graphically in Figure 1.

#### Sociodemographics

Considering that previous research linked male gender, older age, socio-economic disadvantage (i.e., lower education and lower socio-economic status), and rural residency with lower levels of climate change concern (Milfont et al., 2015; McCright et al., 2016; Weckroth and Ala-Mantila, 2022), *we expected these variables to correlate negatively with the “core pro-climate emotions” (climate enthusiasm, anger, anxiety, and sorrow), and positively with climate contempt* (H3). In addition, taking into account that female gender and younger age are related to an increased vulnerability to negative emotions and lower mental wellbeing (Gross et al., 1997; Kring and Gordon, 1998; Aneshensel et al., 2013), we predicted that *they would be positively linked also to climate powerlessness, guilt, and isolation* (H4). Importantly, even though socio-economic disadvantage is a key determinant in lower emotional wellbeing (Gallo and Matthews, 2003; Aneshensel et al., 2013), we did not hypothesize any associations between climate powerlessness, guilt, and isolation, and perceived economic hardship or lower education level since we expected that they might be evened out by heightened climate change skepticism in this socio-demographic segment (Weckroth and Ala-Mantila, 2022).

#### Loneliness

Subjective feelings of loneliness refer to the negative emotional state caused by the perception of being separated from others (Hays and DiMatteo, 1987; Hawkley and Cacioppo, 2010). Emotional loneliness is associated with the experience of negative affect, such as, e.g., sadness, anxiety, or anger, and it is a risk factor for mental disorders (Schultz and Moore, 1984; Hsu et al., 1987; Hawkley and Cacioppo, 2010). Because of the conceptual and empirical overlap between loneliness and climate isolation (Marczak et al., 2023b), as well as due to the links between loneliness and negative affect documented in the literature, we expected that: *There is a positive correlation between loneliness and climate isolation, anxiety, sorrow, guilt, powerlessness and anger* (H5).

#### Alienation

Climate isolation along with climate powerlessness, anger, and its “anti-climate” counterpart, climate contempt, bear conceptual resemblance with the emotional experience of socio-political alienation (Marczak et al., 2023b). Alienation, an important concept in social sciences linked to a number of politically relevant variables (Kalekin-Fishman and Langman, 2015; Schwartz, 2017), refers to strong feelings around an individual’s sense of separation from the society and its values as well as a sense of lack of influence over socio-political events (Seeman, 1959; Thompson and Horton, 1960). Because of this conceptual overlap, we hypothesized that: *There is a positive correlation between alienation and climate isolation, powerlessness, anger, and contempt* (H6).

Although loneliness and alienation bear some degree of similarity, it is important to emphasize that they differ in their nature and focus. Loneliness addresses primarily individual social isolation - the perception of being separated from others, resulting in a negative emotional state often leading to psychological distress. Alienation, on the other hand, extends to broader societal and political disconnection, not just from other people but from society, its values, and socio-political events. The differing hypotheses reflect the unique relationships of these constructs with climate-emotions, depending on whether the focus is on the distress caused by the subjective sense of feeling lonely or broader societal and political disconnection.

#### Willingness to sacrifice for the environment

The extent to which one is willing to renounce one’s own immediate self-interests in favor of the well-being of the natural environment has been strongly positively linked to pro-environmental behavior (Iwata, 2002; Davis et al., 2011). Therefore, it is potentially an important aspect of fostering pro-climate behavior. To conceptually replicate the positive and negative associations of “pro-climate emotions” and climate contempt, respectively, with pro-climate engagement, we hypothesized that: *Willingness to sacrifice for the environment is positively correlated with climate enthusiasm, anger, anxiety, and sorrow, and it correlates negatively with climate contempt* (H7).

#### Environmental activism

Mass engagement in efforts to bring about political or social change is a key driver of sustainability transition (Roser-Renouf et al., 2014; Wallis et al., 2021). Such efforts can be defined as deliberate civic behavior addressing systemic roots of environmental issues and advocating collective action for sustainability transition (Alisat and Riemer, 2015). Empirical research demonstrated that environmental activism can be divided into two categories: ‘leadership actions’, in which a person takes responsibility for organizing an action such as a protest or a boycott, and ‘participatory actions’ such as taking part in a protest or informing others about environmental issues (Alisat and Riemer, 2015).

Emotional engagement has been recognized as an important factor encouraging environmental activism (Roser-Renouf et al., 2014), with group-based guilt (Rees and Bamberg, 2014; Haugestad et al., 2021) and anger (Van Zomeren et al., 2004) being the most important discrete emotions associated with civic engagement. However, climate guilt and anger in the ICE capture predominantly the individual experience of these emotions. For this reason, we assumed, in line with the previous findings regarding pro-climate emotions (Marczak et al., 2023a), that *environmental activism, both in terms of participatory actions and leadership actions, is correlated positively with the core pro-climate emotions such as climate enthusiasm, anger, anxiety and sorrow, and it is negatively associated with climate contempt* (H8).

## Method

### Participants and procedure

The participants in this study were divided into three groups according to their country of residence. We collected original data in two countries - Norway and Ireland, and, for the analysis of measurement invariance, we also used secondary data from Poland described in Marczak et al. (2023a). Group 1 comprised 831 residents of Norway, group 2 were 775 residents of Ireland, and group 3 were 319 residents of Poland. All study participants were quota sampled from the general population according to their level of concern about climate change. Prior to data collection, the distributions of climate change concern within the studied populations were estimated by the contracted panel-data provider based on an omnibus screening conducted on panel-based samples. Such sampling strategy was aimed at reaching a balanced distribution of different levels of concern about this issue. Moreover, it allowed for collecting data in Norway and Ireland comparable to the secondary data from Poland, where this sampling strategy was employed in the first place (see Marczak et al., 2023a for details).

After a data quality check and anonymization, 491 responses remained in the sample from Norway, 485 in the Irish sample, and 300 in the sample from Poland. Data quality check and study procedure for Poland is described in the paper from which we sourced the data (Marczak et al., 2023a). The details on data quality check for Norway and Ireland, which follow the same criteria as in the study by Marczak et al. (2023a), are presented in the subsection below. The socio-demographic characteristics of all samples are presented in Table 1.

**Table 1:** Socio-demographic characteristics of study participants.

	Sample 1–Norway (*n* = 491)	Sample 2–Ireland (*n* = 485)	Sample 3–Poland (*n* = 300)
% Women	50	61	59
Mean age (SD)	46.77 (14.61)	42.55 (13.47)	40.30 (14.63)
% Urban population	72	74	80
Education			
% Primary Education	6	2	4
% Secondary education or vocational training	47	42	47
% University/College degree and higher	46	55	49
Perceived SES			
% “Living comfortably on present income”	29	25	10
% “Coping on present income”	43	46	71
% “Finding it difficult on present income”	23	22	19
% “Finding it very difficult on present income”	6	7	0
Climate change concern			
% “Not at all concerned”	13	8	6
% “Not very concerned”	14	10	6
% “Somewhat concerned”	33	28	39
% “Very concerned”	31	31	42
% “Extremely concerned”	10	24	7

Due to rounding the numbers to integers, some data reported in percentage sums up to 100 ± 1.

We aimed to collect at least 300 responses in each country to ensure that the requirements for the minimum sample size for factor analysis (Tabachnick et al., 2007) were met. In addition, power analysis conducted with the pwr R package (Champely, 2020) indicated that, at *α* = 0.05, with the final sample sizes in Norway and Ireland, we were able to detect a true weak correlation (*r* = 0.15) with the power of 0.92 and 0.91, respectively.

The participants in Norway and Ireland completed the study using an online platform developed for the purpose of the broader Climate Emotions project to enhance defining research tasks without relying on browser-based configuration tools. The study was advertised by the contracted panel data provider, and those willing to participate and meeting the screening criteria were provided with a link to the online procedure. The participants first read the description of the aims of the study and provided their informed consent. They were also informed that the procedure included control questions verifying their attention (e.g., “To convince us that you are reading this, please, just mark the option “Strongly disagree”). Next, they evaluated the climate emotions items presented in a random order. In the next block, they completed additional scales for the assessment of correlates of climate change emotions. The measures used in this part of the procedure differed between the two samples (see below). The order of the scales was randomized, and within each scale the items were presented in a randomized order too. In the last block, participants answered questions about their socio-demographics. Participants were compensated with points that could be exchanged for payment according to their preference and the panel’s standard criteria.

### Materials

We created slightly different versions of the survey for Norway and Ireland. The two versions presented below differed only slightly in terms of the measures used to broaden the nomological scope of climate emotions. On top of the questionnaires used in both countries to measure climate emotions, perceptions, policy support, and socio-demographics, in the survey in Norway, we also inspected loneliness and willingness to sacrifice for the environment, whereas in Ireland, we added the measures of alienation and environmental activism. This decision was driven by practical considerations. Firstly, our objective was to test all the hypotheses without risking putting too much response burden on the participants. Secondly, this approach allowed us to maximize the statistical power of the subsequent correlational analyses. Finally, the utilization of different survey versions is a well-established strategy in questionnaire validation studies, as demonstrated by, e.g., Milfont and Duckitt (2010). By doing so, we aimed to ensure robust results within the constraints of available resources. To obtain the Norwegian-language versions of the measures used in this study, we used the translation/back-translation procedure. In addition, the translation procedure for the ICE is described in detail below. The internal consistency values of climate emotions and all the other variables are reported in Tables 2 and 3, respectively.

**Table 2:** Parameter estimates from confirmatory factor analyses and internal consistencies of the scales (Cronbach’s α and Raykov’s *ρ*).

Factor name (*α*, *ρ*)	Indicator	Norway				Ireland			
		*B*	SE	*z-*value	*β*	*B*	SE	*z-*value	*β*
Climate anger (NOR: *α* = 0.91, *ρ* = 0.91; IRL: *α* = 0.89, *ρ* = 0.89)	I feel angry that the political and economic system that we live in harms the climate.	1.00			0.84	1.00			0.81
	I am outraged that politicians allowed climate change to come this far.	1.10	0.04	25.61	0.87	1.1	0.06	18.45	0.82
	I feel outraged at corporations that harm the climate.	0.96	0.05	20.92	0.80	0.95	0.06	16.56	0.78
	I feel anger when I think of politicians who delay efforts to mitigate climate change.	1.09	0.04	24.93	0.87	1.11	0.06	19.51	0.84
Climate contempt (NOR: *α* = 0.84, *ρ* = 0.85; IRL: *α* = 0.84, *ρ* = 0.84)	It annoys me to watch people succumb to climate hysteria.	1.00			0.55	1.00			0.69
	I am annoyed by the constant publicity around climate change.	1.85	0.16	11.74	0.89	1.28	0.08	16.35	0.82
	I am bored of hearing about climate change.	1.87	0.16	11.67	0.91	1.39	0.08	17.61	0.87
	I am surprised that people experience strong emotions in connection with climate change.	1.14	0.11	10.46	0.63	0.92	0.07	12.65	0.62
Climate enthusiasm (NOR: *α* = 0.83, *ρ* = 0.83; IRL: *α* = 0.80, *ρ* = 0.80)	The increasing public engagement with climate change gives me hope.	1.00			0.84	1.00			0.78
	I believe that there are emerging solutions that will allow us to stop climate change.	0.77	0.05	14.71	0.68	0.81	0.08	9.87	0.63
	Concrete actions for the climate allow me to be optimistic about the future.	0.73	0.06	13.22	0.66	0.84	0.07	12.36	0.67
	Social mobilization in the fight against climate change makes me feel that together we can achieve this goal.	0.86	0.05	18.92	0.77	1.01	0.07	14.64	0.76
Climate powerless- ness (NOR: *α* = 0.72, *ρ* = 0.73; IRL: *α* = 0.65, *ρ* = 0.66)	I feel confused about what I can do to reduce climate change.	1.00			0.56	1.00			0.51
	I am overwhelmed by how many aspects of life would need to be changed to limit climate change.	1.21	0.12	10.06	0.71	1.06	0.12	8.63	0.56
	As an individual, I feel powerless with little agency over what happens with the climate.	0.88	0.12	7.10	0.49	0.93	0.12	7.81	0.50
	I feel helpless when I think of how difficult it is to live in a climate-friendly way.	1.36	0.13	10.17	0.76	1.40	0.14	9.83	0.72
Climate guilt (NOR: *α* = 0.90, *ρ* = 0.90; IRL: *α* = 0.88, *ρ* = 0.88)	I have a guilty conscience about not doing enough to mitigate climate change.	1.00			0.84	1.00			0.84
	It upsets me that I have a big negative impact on the climate.	0.87	0.04	19.64	0.77	0.82	0.04	20.31	0.72
	I feel guilty that my lifestyle contributes to climate change.	1.01	0.04	27.27	0.85	1.00	0.04	28.00	0.86
	I am angry at myself for not doing enough to limit my negative impact on the climate.	0.96	0.03	27.95	0.86	0.93	0.04	24.71	0.79
Climate isolation (NOR: *α* = 0.85, *ρ* = 0.85; IRL: *α* = 0.77, *ρ* = 0.77)	I feel like one of the few people who actually understand what climate change entails.	1.00			0.67	1.00			0.55
	I feel lonely because most of the people around me do not care about climate change as much as I do.	1.20	0.08	15.97	0.81	1.22	0.14	9.00	0.70
	I feel lonely because it’s difficult to talk about my climate change concerns with other people.	1.22	0.08	15.85	0.82	1.38	0.13	10.72	0.79
	I feel alienated because society considers concern for climate change as something strange.	1.12	0.08	14.20	0.77	1.10	0.12	9.31	0.65
Climate anxiety (NOR: *α* = 0.85, *ρ* = 0.85; IRL: *α* = 0.77, *ρ* = 0.77)	Thinking about climate change makes me fear for the future of our children.	1.00			0.86	1.00			0.86
	I am overwhelmed by the awareness of the approaching climate disaster.	0.87	0.04	24.19	0.80	0.69	0.04	16.45	0.61
	Everything seems uncertain because of climate change.	0.77	0.04	21.60	0.76	0.71	0.05	14.39	0.64
	I fear how climate change will affect me and my loved ones.	0.92	0.03	28.04	0.84	0.98	0.03	33.53	0.85
Climate sorrow (NOR: *α* = 0.87, *ρ* = 0.87; IRL: *α* = 0.88, *ρ* = 0.88)	The thought of so many species going extinct under the pressure of climate change fills me with sorrow.	1.00			0.74	1.00			0.82
	The thought that the world I know is disappearing forever because of climate change makes me sad.	1.18	0.07	17.52	0.84	1.09	0.05	21.48	0.85
	I feel sorry about the possibilities we are losing forever because of climate change.	1.08	0.06	18.28	0.84	0.95	0.05	17.96	0.77
	I am sad that so many living creatures suffer because of climate change.	0.95	0.05	19.07	0.73	0.88	0.05	18.40	0.77

**Table 3:** Descriptive statistics of study variables.

Variable	*M*	SD	Skewness	Kurtosis	*α*
Norway					
Climate anger	3.31	1.11	−0.43	−0.58	0.91
Climate contempt	2.88	1.10	0.12	−0.84	0.84
Climate enthusiasm	3.24	0.85	−0.69	0.32	0.83
Climate powerlessness	3.09	0.84	−0.26	−0.13	0.72
Climate guilt	2.62	1.02	0.08	−0.78	0.90
Climate isolation	2.36	0.94	0.50	−0.18	0.85
Climate anxiety	3.10	1.03	−0.45	−0.58	0.85
Climate sorrow	3.56	1.00	−0.76	0.08	0.87
Pro-climate perceptions	4.88	1.40	−0.65	−0.11	0.79
Mitigation policy support	4.99	1.43	−0.75	0.21	0.87
Loneliness	2.05	0.69	0.47	−0.58	0.89
Willingness to sacrifice	5.91	1.99	−0.49	−0.17	0.94
Ireland					
Climate anger	3.90	0.97	−0.99	0.55	0.89
Climate contempt	2.68	1.09	0.43	−0.75	0.84
Climate enthusiasm	3.66	0.78	−0.85	0.85	0.80
Climate powerlessness	3.29	0.80	−0.35	0.17	0.65
Climate guilt	3.11	1.04	−0.48	−0.58	0.88
Climate isolation	2.70	0.91	0.24	−0.41	0.77
Climate anxiety	3.56	0.97	−0.81	0.14	0.77
Climate sorrow	4.00	0.97	−1.25	1.16	0.88
Pro-climate perceptions	5.24	1.27	−1.01	1.07	0.76
Mitigation policy support	5.28	1.27	−0.95	0.86	0.84
Alienation	2.93	0.54	−0.32	0.14	0.71
Leadership action	1.48	0.86	2.32	4.89	0.94
Participatory action	2.11	0.92	1.06	0.54	0.91

### Materials used across countries

#### Climate change emotions

Participants evaluated 32 ICE items about their emotional experience of climate change across eight scales described in the introduction (Marczak et al., 2023a). They marked their responses on a 5-point Likert-scale from “strongly disagree” (1) to “strongly agree” (5). The higher the score, the more the person identified with experiencing a given climate emotion.

The items were translated by professional translators from Polish into Norwegian and English. Next, the translations were carefully compared to the original version by the first author fluent in Polish, Norwegian, and English, who made adjustments to keep the meaning as close as possible to the original scale. Next, the items were back-translated by another pair of professional translators. The original and back-translated Polish versions were then compared and further minor adjustments were made to the discrepant items. In the last step, the translated items were presented to two native Norwegian speakers, and two native English language speakers, respectively, who suggested stylistic corrections, constrained by keeping the meaning of the items as close as possible to the original ones. The translated/back-translated Norwegian and English language versions of the ICE are available in the online repository dedicated to storing the current and future various language versions of the instrument: https://osf.io/rfn6m/. In addition, all the ICE items in English are listed in Table 2.

#### Climate change perceptions

We assessed people’s perception of the reality and anthropogenic causes of climate change, as well as the perceived valence, spatial distance, and temporal distance of consequences of climate change using the 5-item version of the Climate Change Perceptions Scale (Van Valkengoed et al., 2021). Responses were marked on a 7-point Likert-scale from “strongly disagree” to “strongly agree.” Additionally, the item related to the perception of the realness of climate change retained the response option ‘I do not believe climate change exists,’ mirroring the original version of the scale. Another example item from this scale is: “Climate change will bring about serious negative consequences.” The higher the score, the more the person believed that climate change is a real and human-made problem that will bring about negative consequences not far in time and space.

#### Mitigation policy support

We assessed participants’ support for policies aimed at mitigating climate change relying on five items developed by Van Valkengoed et al. (2021). The items were answered using a 7-point Likert-scale from “strongly oppose” to “strongly support.” The measure includes items such as: “Using public money to subsidize renewable energy such as wind and solar power” and “Setting national targets to reduce carbon emissions.” The higher the score, the more the participant supported climate change mitigation policies.

#### Socio-demographic and background information

Participants indicated their gender, year of birth, educational attainment, area of residence, perceived socio-economic status, and marked their level of concern about climate change on a scale from 1 to 5 (from “not at all concerned” to “extremely concerned”).

### Additional materials used in Norway

#### Loneliness

We gauged participants’ disposition to feelings of being cut off or separated from others using the 8-item short version of the UCLA Loneliness Scale (ULS-8, Hays and DiMatteo, 1987). We recorded participants’ responses on a 4-point Likert scale (‘1’ - “never,” ‘2’ - “rarely,” ‘3’ - “sometimes,” ‘4’ - “often”). The higher the score, the more lonely the person felt. Example items from this scale are: “People are around me but not with me” or “I feel isolated from others.”

#### Willingness to sacrifice for the environment

To gauge whether participants were willing to sacrifice their own needs for the sake of the environment, we used a 5-item scale developed by Davis et al. (2011). The 9-point response scale ranged from “do not agree at all” to “agree completely.” The higher the score, the more the participant was willing to renounce their own self-interest in favor of the natural environment. The scale comprises items such as, e.g., “I am willing to give up things that I like doing if they harm the natural environment” or “Even when it is inconvenient to me, I am willing to do what I think is best for the environment.”

### Additional materials used in Ireland

#### Alienation

Participants’ feelings of alienation were assessed using the 6-item short version of the Mcclosky and Schaar’s (1965) alienation scales adapted from Rudnev et al. (2018). Participants responded using a 4-point scale from “strongly disagree” to “strongly agree.” Example items from this scale are: “Politicians do not care what people like me think” or “Nowadays things are so complex that you sometimes do not know what is going on.” The higher the score, the higher the participant’s alienation.

#### Environmental activism

Participants’ level of engagement in civic environmental actions in terms of participatory actions and leadership actions was assessed using the 18-item environmental action scale (Alisat and Riemer, 2015). The participants marked their responses on a 5-point scale from “never” through “sometimes” to “frequently.” An example item from the participatory actions subscale is “I took part in a protest/rally about an environmental issue,” and an example item from the leadership actions subscale is “I organized a community event which focused on environmental awareness.” The higher the score, the more the person was involved in the given type of civic pro-environmental action.

### Data quality check

To ensure data quality, we evaluated two key aspects of online behavioral data quality – honesty and attention (Peer et al., 2022). As such, we defined the following criteria: (1) the participants’ responses regarding gender and age had to be consistent with their responses to the same questions in the screening survey, and (2) they were required to respond correctly to all three attention check questions. Overall, 59% of responses were retained for further analysis in the data from Norway, and 63% in the data from Ireland.

A possible explanation for these proportions being rather low is that the structure of compensation in our study was based on the completion of the entire procedure rather than the quality of individual responses. What is more, the relative high socio-economic prosperity of the Norwegian and Irish population might have led to a scenario where participant remuneration, while consistent with standard practices, may not always have served as a sufficient motivating factor to elicit honesty and attention in responses to survey questions. Nevertheless, despite the reduction in the volume of responses included in the analyses, the data quality check ensured that the retained data accurately capture the intended constructs. This, in turn, enhances the overall validity and reliability of our findings, especially against the backdrop of often compromised quality of online behavioral data and the lack of universal adoption of data quality checks from online panels (Arndt et al., 2022).

### Data analysis

In line with the recommendations for the structure stability and equivalence testing in the literature (Byrne, 2008), we began our analyses with testing the validity of the hypothesized factor model of the ICE for each group separately. We then moved on to imposing increasingly restrictive constraints on the parameters of interest for all groups at the same time to establish whether the instrument under investigation is equivalent across the studied samples. Finally, we conducted correlational analyses to test the hypotheses regarding the nomological scope of climate emotions.

The distribution of the data from Norway and Ireland was evaluated using Mardia’s test for multivariate skewness and kurtosis, and the Shapiro–Wilk test for univariate normal distribution (Byrne, 2001). In both cases, the data departed from multi- and univariate normal distribution. Therefore, the confirmatory factor models were estimated with maximum likelihood estimation with robust standard errors and Satorra-Bentler scaled test statistic (Brown, 2015). The datasets were large enough to warrant high statistical power (*N:q* ratio was 15:1 for both Norway and Ireland samples). The specification of the models was based on the original 32-item model developed and validated by Marczak et al. (2023a).

We assumed after Hu and Bentler (1999) that reasonably good fit is established by a model when the scaled CFI and TLI values are close to 0.95 or greater, RMSEA values are close to 0.06 or below, and SRMR values are close to or below 0.08. To evaluate model comparisons in the investigation of measurement equivalence, we adopted the recommendations that the change of (scaled) CFI between the compared models should not exceed the value of 0.01 (Cheung and Rensvold, 2002).

All the data analyses were conducted using the R Statistical Software (v4.1.2; R Core Team, 2021). Factor analyses were performed using the lavaan package for R (Rosseel, 2012). All datasets, data cleaning, data analysis scripts, as well as the results not outlined here are available in the online repository: https://osf.io/r8g6h.

## Results

### Confirmatory factor analyses

In both samples, the original 32-item model demonstrated excellent fit [for Norway: *scaled χ*^2^(436) = 816.53, *p* < 0.001, *SRMR* = 0.05, *scaled RMSEA* = 0.042 (90% *CI* = 0.038, 0.046), *scaled TLI* = 0.95, *scaled CFI* = 0.96; for Ireland: *scaled χ*^2^(436) = 718.92, *p* < 0.001, *SRMR* = 0.05, *scaled RMSEA* = 0.037 (90% *CI* = 0.032, 0.041), *scaled TLI* = 0.95, *scaled CFI* = 0.95]. Factor loadings in both models were above the customary threshold of 0.4 (Brown, 2015).

Internal consistency values (Cronbach α and Raykov’s ρ) ranged between good and excellent for all scales except climate powerlessness in the model for Ireland, which presented weak yet acceptable internal consistency (*α* = 0.65, *ρ* = 0.66). Table 2 presents the details of confirmatory factor models for both countries.

### Measurement equivalence

We estimated and compared increasingly constrained CFA models with each other across Norway, Ireland, and Poland. The fit of the factor structure was reasonably good when the estimates were calculated individually for each group [*scaled χ*^2^(1308) = 2055.112, *p* < 0.001, *SRMR* = 0.05, *scaled RMSEA* = 0.037 (90% *CI* = 0.034, 0.039), *scaled TLI* = 0.95, *scaled CFI* = 0.96; all factor loadings were > 0.40]. Good fit of the configural model provides evidence that the items load on the same factors for all groups. When constraining the factor loadings of the items to be equal across groups, *Δ scaled CFI* was 0.005, lending evidence for the metric equivalence, i.e., that there are no significant differences across the samples in the perception and interpretation of the content of each item. In the next step, we also constrained the intercepts to be equal across the three countries (i.e., scalar equivalence). This time, *Δ scaled CFI* was 0.013, suggesting notable differences in scale properties across groups. Using the Lagrange Multiplier Test (Byrne, 2013), we identified the parameters with significant negative impact on the model fit. There were significant cross-country differences in the intercepts for the items “I am overwhelmed by the awareness of the approaching climate disaster” from the climate anxiety scale and “I feel alienated because society considers concern for climate change as something strange” from the climate isolation scale. We released the constraints on these two items in a stepwise manner, which allowed us to establish partial scalar equivalence (*Δ scaled CFI* = 0.009).

In addition, we also demonstrated that using the Inventory of Climate Emotions, it is possible, with very few deviations from the original formulation of the scale, to measure the same constructs across men and women, age groups (millennials, generation X, and baby boomers), and groups reporting different levels of climate change concern (three levels - low, medium, and high concern). The results can be found in the supplementary material available in the accompanying OSF repository.^2^

### Nomological span

In the first step, we investigated descriptive statistics and Cronbach’s alpha coefficients. The values for all scales are presented in Table 3. The internal consistencies of the scales were above the acceptable level.

**Figure 2:**
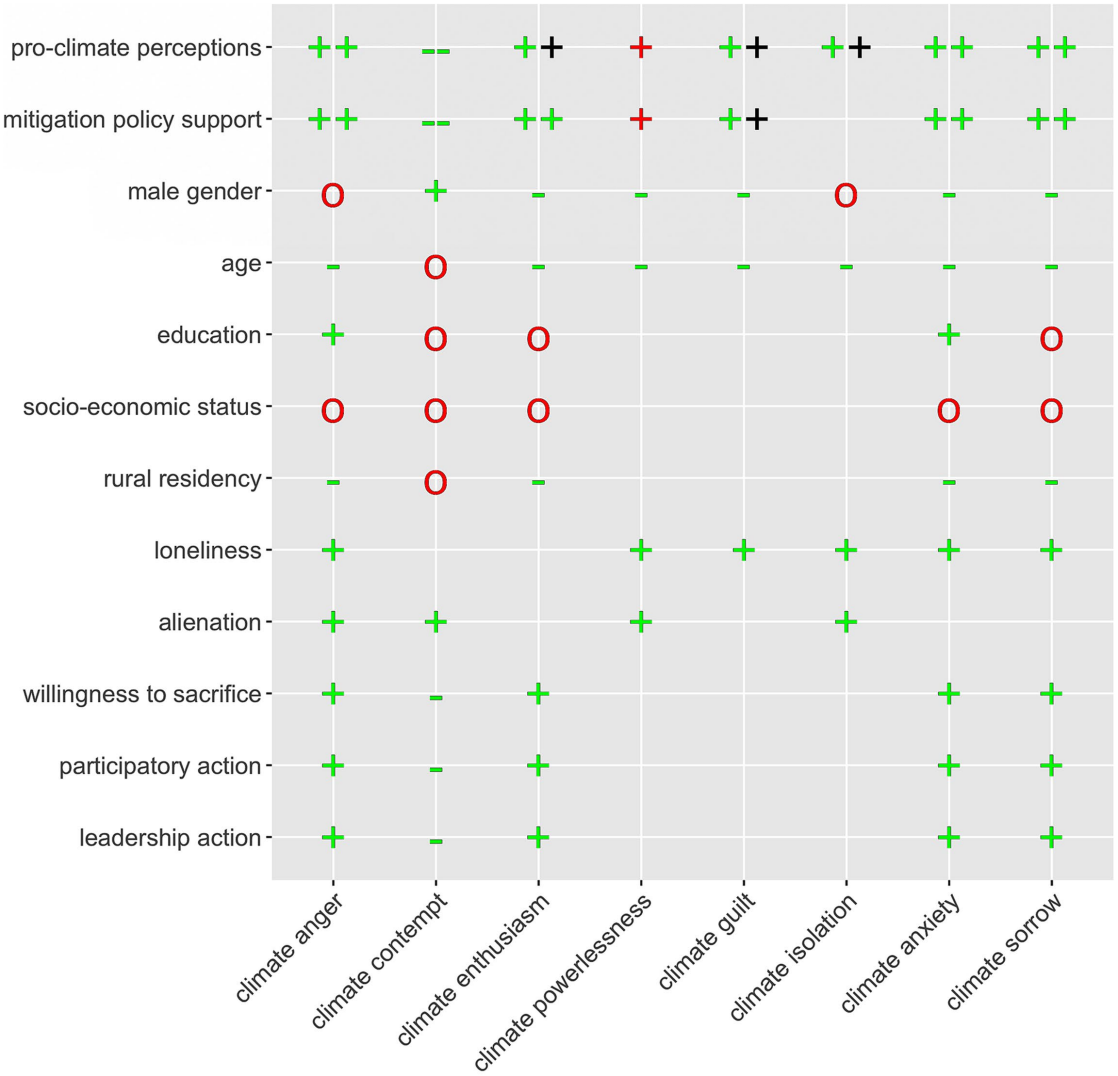
Graphical summary of the results in light of the hypotheses regarding the nomological network of climate emotions. Symbols marked in green signify empirical confirmation of the hypothesized relationship in at least one country. The red color signifies that the hypothesized association was not confirmed empirically, and the value of the symbol indicates the nature of the empirical relationship. In three cases, the associations were stronger than expected, which is marked with an additional symbol in black.

In the next step, we computed Spearman’s rank correlations between climate emotions and theoretically related variables. They are reported in Table 4. Figure 2 sums up the results of correlational analysis in light of the hypotheses. Importantly, we directly replicated the results obtained by Marczak et al. (2023a), however, the correlations hypothesized to be of moderate magnitude, turned out to be stronger than expected. In addition, we observed unexpected positive correlations between climate powerlessness and pro-climate perceptions, as well as mitigation policy support. All other partially confirmed hypotheses were related to the partly exploratory associations between climate emotions and sociodemographic variables.

**Table 4:** Spearman r_s_ correlation coefficients between climate change emotions and theoretically related variables.

	Anger	Contempt	Enthusiasm	Powerlessness	Guilt	Isolation	Anxiety	Sorrow
Variables assessed in both countries								
Pro-climate perceptions Norway	0.65***	−0.69***	0.38***	0.30***	0.48***	0.26***	0.64***	0.69***
Pro-climate perceptions Ireland	0.64***	−0.66***	0.33***	0.19***	0.46***	0.24***	0.59***	0.66***
Mitigation policy support Norway	0.65***	−0.61***	0.49***	0.33***	0.49***	0.30***	0.59***	0.63***
Mitigation policy support Ireland	0.60***	−0.50***	0.43***	0.17***	0.39***	0.29***	0.52***	0.56***
Male gender Norway	**−0.08**	**0.09**	−0.12**	−0.12*	−0.12**	**0.01**	−0.11*	−0.15***
Male gender Ireland	**−0.09**	0.25***	**−0.08**	−0.14**	−0.15**	**0.04**	−0.13**	−0.26***
Age Norway	−0.17***	**0.04**	−0.14**	−0.30***	−0.30***	−0.18***	−0.18***	−0.16***
Age Ireland	−0.13**	**−0.04**	−0.10*	−0.24***	−0.20***	−0.29***	−0.21***	−0.13**
Education Norway	**0.09**	**−0.06**	**0.02**	−0.02	0.01	−0.02	**0.04**	**0.07**
Education Ireland	0.11*	**−0.06**	**0.04**	0.08	0.11*	0.15**	0.13**	**0.06**
Perceived SES Norway	**0.04**	**−0.02**	**−0.02**	−0.02	0.00	0.03	**0.01**	**0.01**
Perceived SES Ireland	**0.07**	**0.00**	**−0.05**	−0.01	0.00	−0.04	**−0.01**	**0.05**
Rural residence Norway	−0.15***	**0.03**	−0.13**	−0.17***	−0.16***	−0.10*	−0.15***	−0.12**
Rural residence Ireland	**−0.08**	**−0.04**	**−0.06**	−0.04	−0.08	−0.17***	**−0.05**	**−0.01**
Variables assessed only in Norway								
Loneliness	0.15***	0.01	0.01	0.35***	0.25***	0.26***	0.16***	0.09*
Willingness to sacrifice	0.58***	−0.48***	0.44***	0.20***	0.47***	0.35***	0.58***	0.58***
Variables assessed only in Ireland								
Alienation	0.13**	0.22***	0	0.27***	0.11*	0.14**	0.11*	0.09*
Leadership action	0.38***	−0.26***	0.37***	0.12**	0.43***	0.40***	0.46***	0.36***
Participatory action	0.21***	**−0.02**	0.22***	0.12**	0.27***	0.38***	0.28***	0.16***

*p < 0.05; **p < 0.01; ***p < 0.001. Correlations included in the hypotheses are marked in bold.

Namely, we only partly confirmed H3, as contrary to our predictions, we found no correlations between male gender and climate anger, and isolation. What is more regarding H3, against our predictions, age and rural residency did not correlate positively with climate contempt, and economic disadvantage was only vaguely correlated with climate emotions. H4 was mostly confirmed with one exception - female gender did not correlate positively with climate isolation.

## Discussion

Emotional responses to climate change have important implications for various aspects of sustainability, including pro-climate engagement as well as health and wellbeing (Clayton and Karazsia, 2020; Brosch and Steg, 2021; Stanley et al., 2021; Pihkala, 2022; Sangervo et al., 2022; Marczak et al., 2023a,b). From this perspective, reliable measurement of these responses is critical for well-grounded research that can inform sustainability transition efforts (Ijzerman et al., 2020; Wallis et al., 2021). In this paper, we inspected evidence for the cross-cultural validity of the Inventory of Climate Emotions (ICE), a measure of multiple emotions experienced in relation to climate change proposed by Marczak et al. (2023a).

Overall, we demonstrated robust psychometric properties of the Norwegian and English language versions of the ICE by showing that the instrument measured the same constructs across Norway, Ireland, and Poland. Furthermore, we found support for most of the hypothesized links regarding the nomological span of climate emotions. Below, we discuss the results of factor analyses and hypotheses testing in more detail. We finish by reflecting on the limitations and implications of this work for future research.

### Discussion of the cross-cultural structure and equivalence of the ICE

Confirmatory factor analyses performed separately on data from Norway and Ireland lent strong support for the cross-cultural stability of the 8-factor structure of the ICE. In addition, internal consistency analyses yielded good to excellent values for each scale, except for climate powerlessness in the sample from Ireland, which, nevertheless, demonstrated acceptable values. These results speak strongly in favor of the psychometric validity of seven of the ICE scales, yet they cast doubt on the climate powerlessness scale.

This scale demonstrated rather low internal consistency values already in the initial scale validation in Poland (Marczak et al., 2023a). Looking at the values of average variance extracted across our confirmatory factor analyses in the two countries (presented in detail in the supplementary material), we found evidence for convergent validity (*AVE* > 0.40) for all scales but climate powerlessness in the CFA model for Ireland (0.33). Echoing Marczak et al. (2023a), we recommend caution with the use of this scale as it might not be a reliable indicator of the feelings of helplessness about one’s agency to address climate change.

In the next step, through a series of tests, we demonstrated the equivalence of the meaning of climate emotions across Norway, Ireland, and Poland. Specifically, we demonstrated that the basic organization of climate emotions as postulated by the ICE is supported in the three countries (configural equivalence), that each item contributes to the specified latent construct to a similar degree across groups (metric equivalence), and that, with the exception for only two items, mean differences in the eight latent climate emotions capture all mean differences in the shared variance of the observable indicators (partial scalar equivalence; Putnick and Bornstein, 2016).

The identified non-invariance of the intercepts for two items - “I am overwhelmed by the awareness of the approaching climate disaster” (climate anxiety) and “I feel alienated because society considers concern for climate change as something strange” (climate isolation) - indicates that, when taking out the part that is explained by the respective latent variables, these items have different mean scores across countries, while the means of other items do not differ. The source of non-equivalence of these items might be a slightly different understanding of the wording or that there is something unique captured in these items that is not covered by the latent variable but differs between the countries.

One plausible explanation for this non-invariance could be related to the perception of the term ‘overwhelm’ in the context of climate anxiety. It is possible that some individuals may interpret ‘overwhelm’ as such an intense form of anxiety or distress that they do not align it with the other items, even when they acknowledge the importance of climate change. This could lead to differential responses and contribute to the observed non-invariance. Additionally, the wording of the item on climate isolation, particularly the terms “society” and “strange,” may play a significant role. It is essential to consider the socio-cultural context and the role of culture in shaping perceptions of climate change as it is possible that individuals in different countries perceive their societies differently in terms of their climate attitudes and social rules guiding the expression of concern about climate change (Norgaard, 2006; Head and Harada, 2017).

Importantly, partial scalar equivalence is established when the majority of items on the factor are invariant (Vandenberg and Lance, 2000) which is the case in our research. Thus, our results show that using the ICE it is possible to properly measure and compare climate emotions between the studied countries (Byrne, 2008). The analysis of measurement equivalence across genders, age groups, and groups reporting different levels of climate change concern (presented in the supplementary material) provides evidence that the ICE makes it possible to compare emotional responses to climate change in a precise way also across these various groups, lending additional support for the validity of the ICE.

In summary, while the ICE was originally developed in the specific cultural context of Poland, the findings of the current research shed light on its broader applicability. The study’s results demonstrate that the ICE can be effectively utilized also in Norway and Ireland, suggesting its validity beyond the very context in which it was developed. These findings, in conjunction with the high degree of measurement invariance observed across socio-demographic groups, support the notion that the framework of climate emotions as postulated by the ICE holds relevance across various populations, particularly within the European context. Nonetheless, acknowledging the cultural specificity of emotions and the influence of local socio-cultural factors and policy landscapes on climate-related responses, it is crucial to recognize the need for further cross-cultural research, particularly beyond western societies (Henrich et al., 2010) before making stronger claims about the generalizability of the ICE.

### Discussion of the nomological span of the ICE

#### Direct replication

In line with our hypotheses, similarly to the sample from Poland (Marczak et al., 2023a), both in Norway and in Ireland, we found strong positive correlations between the core “pro-climate emotions” (climate enthusiasm, anger, anxiety, and sorrow) and both pro-climate change perceptions and support for climate change mitigation policy. In addition, as expected, we observed strong negative correlations between climate contempt and both pro-climate change perceptions and support for climate change mitigation policy.

Furthermore, in line with the predictions about the sign of the correlations, we found positive associations between pro-climate change perceptions and climate enthusiasm, climate guilt, and climate isolation. The correlation coefficients, however, were larger than the expected moderate values reported previously by Marczak et al. (2023a). Especially climate guilt in Norway and Ireland correlated considerably stronger with pro-climate perceptions than in Poland (Marczak et al., 2023a), based on which we formulated this hypothesis. Likewise, for H2, both in Norway and Ireland, we found notably stronger positive relationships between climate guilt and pro-climate policy support than in the research in Poland from Marczak et al. (2023a). Although exceeding the baseline findings from Poland, these results fall in line with previous research from mostly Western European and North American samples, which demonstrated that experiencing guilt can play an important role in pro-climate engagement (see Shipley and van Riper, 2022 for a meta-analysis).

At the same time, contrary to what we predicted in reference to the findings from Poland (Marczak et al., 2023a), climate powerlessness correlated positively with pro-climate engagement, and it did so moderately/strongly in Norway, and weakly, though at a statistically significant level in Ireland. From this perspective, it is important to consider the close interplay between agency and feelings of powerlessness, a phenomenon that could be intensified by the global nature of climate problems. In such a context, individuals may simultaneously feel motivated to make a difference and yet experience a sense of powerlessness when confronted with the vast and interconnected challenges posed by climate change. In addition, given that the sampling procedures were comparable across these three countries, it is possible that climate emotions, and in our case, especially guilt and powerlessness play a different role for pro-climate engagement depending on cultural factors (Jensen, 2019). Future research could investigate in detail the role of culture in the links between climate emotions and climate change engagement.

#### Correlations with socio-demographics

We found only partial confirmation of the hypotheses regarding the links between climate emotions and socio-demographic variables. First, extending the existing research on the links between gender, age, and climate anxiety (Wullenkord et al., 2021; Whitmarsh et al., 2022), female gender and younger age in our study were rather consistently associated in various ways with pro-climate emotions.

Based on our findings, we confirm that women tend to exhibit a higher degree of emotional engagement with climate change when compared to men. These findings resonate, on the level of affect, with the large body of research showing that, statistically speaking, women not only express greater environmental concern and pro-environmental engagement (e.g., Zelezny et al., 2000; Kennedy and Kmec, 2018; Knight and Givens, 2021), but they also hold more egalitarian and pro-democratic orientations than men (Pratto et al., 2013; Üzümçeker, 2023). This effect has been associated with socialization resulting in higher levels of empathy among women (Milfont and Sibley, 2016; Kamas and Preston, 2021). Future studies could test whether the links between empathy and pro-environmentalism are mediated by emotional engagement.

Interestingly, the correlation coefficients regarding gender and climate emotions were generally larger in Ireland. These findings again shed light on the cross-cultural differences in the links between climate emotions and relevant variables. In the context of gender, the differences may be related to different country-level gender equality levels, with Norway being among the countries most influenced by egalitarian values in that matter, which can affect emotion expression and the very levels of climate change concern (Inglehart and Norris, 2003).

In both countries, we confirmed the predictions that younger age will correlate positively with the core pro-climate emotions and with the powerlessness-guilt-isolation triad pointing to higher emotional engagement and emotional toll of younger generations in reference to climate change issues. These findings corroborate the existing research on the links between climate anxiety and age (Wullenkord et al., 2021; Whitmarsh et al., 2022), and show that the generations that are expected to be particularly vulnerable to climate change are already affected by it on the emotional level. At the same time, contrary to the hypothesis, we did not find positive links between older age and more climate contempt. Our interpretation of this finding is that as much as today’s older people might be not as concerned and emotionally affected by climate change as the younger ones, they are not necessarily more prone to affectively oppose the importance of this topic. Longitudinal studies could track whether the younger cohorts would keep their strong emotional engagement with this topic over the years.

We did not confirm the predictions regarding the negative links between socio-economic disadvantage and spatial marginalization on the one hand, and the core pro-climate emotions on the other. To start with, perceived socio-economic status was not related in any significant way to climate emotions, neither in Norway nor in Ireland. These findings not only do not comply with our hypotheses but they are also different from what was found in this regard in a national survey in Finland, where the perceived financial status of the household differentiated the responses concerning the experience of difficult emotions evoked by climate change (Hyry, 2019). Second, education correlated with only two of the hypothesized emotions–climate anxiety and anger–and it did so only in Ireland. Likewise, although our predictions concerning the associations between climate emotions and residence area were nearly fully confirmed in Norway (with the exception for climate contempt), in Ireland, this hypothesis was not confirmed at all. These results indicate that socioeconomic geography of climate emotions is not as straightforward as we assumed based on the distribution of climate change concern across different socio-demographic groups (Weckroth and Ala-Mantila, 2022).

Here, it is worth noting that socio-economic context influences human perceptions, not only in terms of economic disadvantage but also when it comes to privilege (Grandin et al., 2022). Looking at the socio-economic status at the country level, both Norway and Ireland are among the most affluent countries in the world. It is possible that the country-level wealth, including relative economic security and high educational attainment, resulted in reduced variability in climate emotions when linked with socio-economic variables. Our results pave the way for future studies which could move beyond correlational analysis to look for the roots of cross-country differences in the links between socio-demographic variables and emotional responses to climate change.

#### Correlations with loneliness and alienation

Overall, our hypotheses were confirmed when it comes to subjective feelings of loneliness and alienation. First, the results show that emotional loneliness is primarily conceptually related to climate powerlessness, isolation, and guilt. Interestingly, the strongest correlation magnitude with loneliness was observed for climate powerlessness, suggesting that the feelings of helplessness around the perception of having little agency to fight climate change are considerably linked to the perception of being separated from others. At the same time, the moderate significant associations between emotional loneliness and climate isolation, and guilt can be interpreted in the light of the potential psychological toll of these feelings–both loneliness (Hawkley and Cacioppo, 2010) as well as climate isolation and guilt (Marczak et al., 2023a) were associated with emotional difficulties. Future research and intervention could investigate how increasing people’s sense of community may affect climate emotions in the context of mental health and pro-climate engagement.

Similarly to loneliness, subjective sense of socio-political alienation showed the strongest association with climate powerlessness. These findings corroborate the validity of the climate powerlessness scale because the concept of alienation refers to a sense of lack of influence over socio-political events (Seeman, 1959). The results also shed some light over the nature of climate contempt, confirming our assumption that people who report experiencing this climate emotion are statistically more prone to feel isolated in rejecting the prevailing social values. These findings should not be overlooked given the radicalization charge carried by a sense of alienation (Langman and Kalekin-Fishman, 2013).

#### Conceptual replication

We conceptually replicated the previous findings on the links between specific climate emotions and pro-environmental engagement lending more support to the functional validity of climate emotions. When it comes to willingness to sacrifice for the environment, we found strong effects, showing that the core pro-climate emotions and climate contempt were considerably associated, in a positive and negative way, respectively, with readiness to renounce one’s own immediate self-interests in favor of the natural environment. In addition, we observed that climate guilt, isolation, and powerlessness also showed significant positive associations with willingness to make sacrifices for nature, even though they were not consistently related to pro-climate engagement in previous research (Marczak et al., 2023a) and hence not included in the hypothesis. Here, especially climate guilt was strongly related to the willingness to sacrifice for the environment. Our interpretation, subject to further investigation, is that the combination of desire to do things for the environment and failing to do so increases the feelings of guilt (Ágoston et al., 2022).

Concerning environmental activism, the core pro-climate emotions were especially strongly correlated with leadership action, and less so with participatory action. These findings emphasize the important role of emotional engagement in more effortful and personally risky forms of environmental activism. Speaking of specific emotions, climate anxiety and guilt showed the strongest associations with leadership action, corroborating the existing results (Rees and Bamberg, 2014; Schwartz et al., 2022) but, in case of guilt, going beyond the initial validation of the ICE (Marczak et al., 2023a).

Interestingly, both forms of activism correlated strongly with climate isolation and, in fact, climate isolation was the strongest correlate of participatory action. We did not expect to find such a link. Our interpretation of it is that even though activism may give an individual a sense of community with a group of people endorsing similar values (Marczak et al., 2023b), it may come at the cost of exacerbating the feelings of being separated from the rest of the population, who may be perceived as ignoring the need for more decisive climate action. Such a speculative explanation is in line with the well-established psychological phenomenon of pluralistic ignorance which refers to many group members systematically misperceiving what most others think (Katz and Allport, 1931). This systematic bias is widely prevalent when it comes to underestimating the number of people holding pro-climate change beliefs (Geiger and Swim, 2016; Ballew et al., 2020; Geiger et al., 2023).

It is also worth noting that, while climate contempt was moderately negatively related to leadership of environmental activism, its negative link with participation in environmental activism was of negligible magnitude, suggesting that negative emotions around one’s disregard for the issue of climate change might not necessarily lead to less participation in civic action aimed at addressing environmental issues. These findings resonate with research showing that climate change skeptics were still concerned about threats to their local natural environments (Haltinner et al., 2021).

## Limitations and future directions

Some limitations should be considered when interpreting the results. To start with, the samples were not representative of the studied populations, which hinders the generalizability of the results. However, in an attempt to replicate the research on the development of the ICE in Poland (Marczak et al., 2023a), we intentionally quota-sampled from the general populations in Ireland and Norway across different levels of climate change concern. This allowed us to validate the measure of climate emotions across a range of this variable, known to differentiate people’s emotional responses to climate change (Marczak et al., 2023b), and to secure comparable samples for testing measurement invariance.

Another issue is that, though our aim was to reduce response burden, we acknowledge that employing random assessment of constructs across the two studied countries would have been a methodologically more rigorous approach to achieve this goal as it allows for minimizing potential systematic errors. Such an approach should be considered in future studies. In addition, the Climate Perceptions Scale (Van Valkengoed et al., 2021) and the measure of loneliness, ULS-8 (Hays and DiMatteo, 1987), were not validated for use in Norway. As much as these instruments were carefully back-translated, our results regarding these two constructs should be treated carefully.

This research demonstrated a high degree of measurement invariance of the ICE across three countries, and as such it addressed an important goal of science, which is to develop instruments that allow valid comparisons and generalizations. However, it should not be overlooked that both the understanding of one’s emotions and the very perception of climate change are affected by cultural and social factors (Lindquist and Feldman Barrett, 2008; Head and Harada, 2017; Adams, 2021). Since climate vulnerability across the world relates to social groups in different ways (Schlosberg and Collins, 2014), we acknowledge that, beyond quantitative measures, there is space for a more in-depth understanding of emotional experience of climate change with due attention to local processes and meanings.

What is more, while we have strived to provide a comprehensive discussion of our results, it is important to acknowledge the inherent complexity of the subject matter. Cross-cultural differences in emotional responses to climate change can be influenced by various factors, including nuances in the emotional tones conveyed by different questionnaire items. For instance, the climate powerlessness subscale of the ICE includes items such as confusion and feeling overwhelmed, alongside helplessness and powerlessness. Despite the confirmed psychometric quality of this scale, it is crucial to recognize that confusion may not always align with feelings of powerlessness, and individuals may experience confusion even when they believe in their capacity to effect change. Similarly, perceptions of being overwhelmed (an indicator of climate anxiety) can vary across cultures, languages, and social norms. Recognizing the complexities involved in interpreting specific climate emotion indicators is essential, particularly in the context of cultural variations in emotional responses and climate change perceptions.

Lastly, the nomothetic span of climate emotions as measured by the ICE was corroborated and extended based on correlational analysis only. Future research could use network analysis to provide a more complex picture of climate emotions and their correlates. Another promising avenue for research would be to employ structural equations modeling to distinguish the unique roles of specific climate emotions in pro-climate engagement and psychosocial impacts of climate change across cultures, as well as ensure the psychometric quality of all the measures by establishing measurement models prior to analyzing the linear relationships.

## Conclusion

The main added value of this research is that it provides evidence for the cross-cultural validity of the ICE in Norway and Ireland. It also corroborates and extends the nomological span of climate emotions by showing that they are differentially linked to climate change perceptions, pro-climate policy support, socio-demographics, loneliness, and alienation, as well as to environmental activism and willingness to renounce one’s own immediate self-interests in favor of the natural environment. We have verified that both positive and negative emotions related to climate change may be linked to pro-environmental attitudes and behaviors. Also, our results show that women and younger people express stronger emotional engagement with climate change.

Importantly, we were the first to investigate, in a psychometrically valid manner, a wide palette of emotional responses to climate change and their correlates in Norway and Ireland. Although this research did not intend to elucidate the differences between these two countries, our results indicate that cultural factors do influence the relationships between climate emotions and theoretically relevant variables. Overall, this paper paves the way for future cross-cultural research on climate emotions and contributes both with validated measurement tools and the R code to perform the analysis, as well as with data on a wide-array of correlates open to more in-depth investigation in line with the suggestions we put forward. Finally, to facilitate prospective cross-cultural work on various emotional responses to climate change, we created a repository for the current and future psychometrically validated various language versions of the ICE publicly accessible under this link: https://osf.io/rfn6m/.

## Data availability statement

The datasets presented in this study can be found at: https://osf.io/r8g6h.

## Ethics statement

The studies involving humans were approved by Norsk Senter for Forskningsdata (NSD). The studies were conducted in accordance with the local legislation and institutional requirements. The participants provided their written informed consent to participate in this study.

## Author contributions

MM: conceptualization, methodology, software, validation, formal analysis, investigation, writing – original draft, writing – review and editing, visualization, project administration, and funding acquisition. MW: investigation, data curation, writing – review and editing, project administration, resources, and validation. BK: resources, and data curation. AM: writing – review and editing, project administration, and funding acquisition. RM: writing – review and editing, and supervision. CK: writing – review and editing, supervision, project administration, and funding acquisition. All authors contributed to the article and approved the submitted version.

## Footnotes

^1^It is worth noting that the deliberate choice of ‘sorrow’ over the more common ‘grief’ ensured a precise focus on the emotional experience linked to the feelings of sadness surrounding irreparable harm to the environment, avoiding the broader connotations of ‘grief’, which can include, e.g., the disruption to daily life and a wider range of feelings beyond sadness, such as anger, guilt, and confusion.

^2^https://osf.io/r8g6h
